# A Retrospective Quality Analysis of External Ventricular Drain Infection Rates Following Stroke Diagnoses and Other Brain Injuries: Comparison of Emergency Room and ICU/OR Setting

**DOI:** 10.7759/cureus.7173

**Published:** 2020-03-03

**Authors:** David Altschul, Mousa K Hamad, Andrew Kobets, Rose Fluss, Christopher Lin, Andre E Boyke, Jinyuan Liu, Rony Thomas, Santiago R Unda

**Affiliations:** 1 Neurological Surgery, Montefiore Medical Center, Bronx, USA; 2 Neurological Surgery, Albert Einstein College of Medicine/Montefiore Medical Center, Bronx, USA

**Keywords:** external ventricular drain, infections, emergency room, intensive care unit

## Abstract

Objective

The purpose of this study was to analyze the incidence of infections in patients following placement of External Ventricular Drain (EVD) in either the Emergency Room (ER) or the Intensive Care Unit (ICU)/ Operating Room (OR) at a single Comprehensive Stroke Center.

Methods

Retrospective analysis of post-procedure infection rates in 710 patients with EVDs placed on site between 2010 and 2018 was performed. We analyzed cases between sex, age, stroke and non-stroke related and further requirement of conversion of the EVD to a ventriculoperitoneal (VP) shunt.

Results

Significant decrease in EVD related infection (ERIs) rates following the shift in EVD placement from ER to ICU/OR (from 13% to 7.7%, p=.03) among all ages, sex and type of brain injury was observed. Furthermore, our data also shows that the rate of conversion of EVDs to VP shunts is independent of the setting where EVD was placed, but increases in patients who develop ERIs. 23.1% of stroke patients that developed an ERI required a conversion to VP shunt while 67.3% of non-stroke patients that developed an ERI required further VP shunt (p<.001) showing that non-stroke EVD patients with infections are more likely to require VP shunt.

Conclusion

This is one of the larger retrospective studies conducted on EVD related infections. ERIs were significantly higher when EVDs were placed in the ER. Moreover, our results highlight the relation between ERIs and further requirement of conversion EVD to VP shunt. These figures highlight the importance of focusing on infection rates, and the implications CSF infection has on the long-term care of patients.

## Introduction

External ventricular drain (EVD), otherwise known as external ventriculostomy, is a neurosurgical procedure that is used for monitoring and treatment of elevated intracranial pressure (ICP) after traumatic brain injury (TBI) as well as primary hydrocephalus or secondary to subarachnoid hemorrhage (SAH), intra-parenchymal hemorrhage (IPH), intra-ventricular hemorrhage (IVH), malignancies and cerebrospinal fluid (CSF) leak [1,2]. Although EVD placement is considered a low-risk procedure, it can carry complications such as hemorrhage, obstruction of the drainage system, and the most common EVD-related infections (ERI) [3-5].

ERI’s rates have been reported to be as high as up to 45% and moreover, the consequent meningitis or ventriculitis are associated with increased morbidity, mortality, hospital stay, prolonged treatment time with antibiotics and costs [2,6]. Therefore, several studies have focused on the prevention of these high infection rates using specialized instruments, including antibiotic-impregnated catheters, and increasing antibiotic prophylactic treatments preoperatively [7-8]. Other authors have suggested possible epidemiological and clinical risk factors for ERIs [6,9-11].

Recently, the location where an EVD can be placed such as Emergency Room (ER), Operating Room (OR), or the Intensive Care Unite (ICU) have also been proposed as a potential predictor for ERIs [1]. However, these results are still inconsistent, possibly due to the fact that most of these studies have been done in relatively small samples. We hypostatize ER setting is an iatrogenic factor that contributes to EVD infections due to its environmental contamination. Thus, our aim is to compare the rates of ERIs considering the environment where EVD was done under in a large sample of patients.

## Materials and methods

For this study, we conducted a large retrospective study and analysis at a quaternary care center in Bronx, New York. With the approval from the Institutional Review Board, this study began with collecting retrospective clinical data from chart reviews under the electronic health record system from a prospective database of all the patients that had undergone an external ventricular drain (EVD) placement from January 2010 to June 2018. Informed consent was waived due to the nature of the research.

Study of the intervention

The primary outcome of this study was to correlate the rates of ERIs with the location (ER or ICU/OR) the EVD was placed. The secondary aims were to assess if higher rates of ERIs are associated with stroke or non-stroke cases, to address if rates of conversion of EVD placements to VP shunt has any correlation with ERIs and to compare rates of EVD placement conversion to VP shunt between stoke patients with ERIs and non-stroke patients with ERIs. For this purpose, we defined ERI as infection occurrence of a positive CSF culture or Gram stain and concomitant occurrence of at least two SIRS criteria (temperature > 38.3 °C or < 36 °C, heart rate > 90 beats/min, respiratory rate > 20 breaths/ min or PaCO2 < 32 mmHg) or occurrence of one of any neurological sign of central nervous system (CNS) infection (nuchal rigidity, headache or changes in mental status) and increased CSF white blood cell count (WBCC).

Patient selection

Patients among all ages that had an EVD placement from January 2010 to June 2018 were included. Exclusion criteria comprised patients with EVD placement as part of a treatment for a preexisting CSF infection. In total, 710 patients were included. 

Intervention: Treatment and procedural management

Selection of patients that underwent EVD placement was done by the neurosurgical team of our institution. EVDs were monitored daily for the complete hospital stay. Patients under 22 years old were treated in the children’s division of our institution while older patients were managed in the adult’s main hospital, in both cases the same neurosurgical team was in charge of the EVD placement. Ventricular-peritoneal shunts (VP shunts) included patients who eventually required a shunt after EVD was placed. Prior to July 2015, all patients that had an EVD placement were in the setting of an Emergency Room (ER). In July 2015, the protocol for EVD placement had been modified to having EVD placement in the Intensive Care Unit (ICU) or in the Operating Room (OR). Indications for obtaining a CSF sample were high clinical or imaging suspicious of nervous system infection and persistent fever. All EVDs placed in our institution were done using non-antibacterial impregnated catheters and antibiotic prophylaxis for Gram-positive coverage was given to the patient before the procedure (ISDA guidelines)[8].

Data collection

According to the aims of our project, we collected factors related to the EVD placement (date of placement, reason for EVD placement, ER or ICU/OR placement), stroke categories which included: 1) Ischemic and 2) Hemorrhagic (SAH, AVM rupture, IPH, IVH and subdural hematoma (SDH)), and non-stroke categories which included: hydrocephalus, tumor bleeding, mass effect, traumatic brain injuries (TBI) and VP shunts failures. Finally, we included the number of patients that required conversion of EVD to VP shunt.

Analysis

SPSS v.24 software was used to perform the statistical analysis. In regard EVD variables, comparisons of the distributions were made by the chi-square (χ2-test) for categorical variables while t-test was used to analyze continuous variables. P<0.05 was considered as statistically significant.

## Results

A total of 710 patients met the inclusion criteria for this study. The general characteristics of our population is shown in Table 1. We had similar distribution of sex (54.1% of males and 45.9% of females) with an average age of 45.9 ± 24.3 years old. The majority of our cohort were stroke patients (398; Ischemic 1.7% and Hemorrhagic 54.83%). Nevertheless, we had a substantial number of non-stroke patients (43.47%) as well. We had complete data of the place of EVD placement (n=710), from which 451 (63.5%) EVDs were placed in the ER while 259 (36.5%) in the ICU/OR. Data for infections were available for 704 patients. Seventy eight (11.1%) patients out of these 704 developed an ERI and 153 (21.6%) patients out of 707 with available records for VP shunt, needed a conversion of EVD to VP shunt.

**Table 1:** Characteristics of patients with EVD placement External ventricular drainage (EVD), subarachnoid hemorrhage (SAH), arterio-venous malformation (AVM,) intraparenchymal hemorrhage (IPH), intraventricular hemorrhage (IVH), subdural hemorrhage (SDH), traumatic brain injury (TBI), emergency room (ER),  EVD-related infection (ERI), intensive care unit/operating room (ICU/OR), ventriculo-peritoneal (VP)

General characteristics of EVD placement		Count	N%
Gender (n=710)	M	384	54.1%
	F	326	45.9%
Age (n=710)	(mean and SD)	45.97	24.32
Stroke (n=398)	Ischemic	12	1.7%
	Hemorrhagic (SAH, AVM, IPH, IVH, SDH)	386	54.83%
Non-Stroke (n=306)	(Hydrocephalus, Tumor bleeding, mass effect, VP shunts failure, TBI)	306	43.47%
Place (n=710)	ER	451	63.5%
	ICU/OR	259	36.5%
ERI (n=704)	No	626	88.9%
	Yes	78	11.1%
Conversion to VP shunt (n=707)	No	554	78.4%
	Yes	153	21.6%

We also collected the rates of EVD related infections (ERIs) for each year from 2010 to 2018 (Table 2). Due to the establishment of a new protocol from July 1st 2015 that changed the standard institutional practice of EVD placement from ER to ICU or OR, we divided 2015 in F2015 (first 6 months of 2015) and L2015 (last 6 months of 2015). Before the introduction of the new protocol, the rates of ERIs varied from 6.1% to 31.3%. ERI’s rates dropped from 12% in the first semester of 2015 to 7.8% in the following 6 months of 2015 with the new protocol, and then a consistent drop of the rates was seen from 2016 with 9.9% until 2018 with 2.9%.

**Table 2:** Rates of EVD infection 2010-2018 External ventricular drainage (EVD), emergency room (ER), intensive care unit/operating room (ICU/OR), First semester of 2015 (F2015), Last semester of 2015 (L2015)

Parameter			EVD infection			
			No		Yes	
			Count (n=626)	N %	Count (n=78)	N %
EVD placement in ER (n=445)	Year	2010	79	85.9%	13	14.1%
		2011	22	68.8%	10	31.3%
		2012	91	87.5%	13	12.5%
		2013	77	93.9%	5	6.1%
		2014	74	87.1%	11	12.9%
		F2015	44	88.0%	6	12.0%
EVD placement in ICU/OR (new protocol) (n=259)		L2015	47	92.2%	4	7.8%
		2016	91	90.1%	10	9.9%
		2017	67	93.1%	5	6.9%
		2018	34	97.1%	1	2.9%

Regarding the relation between the environment where EVD was placed and infections, sex distribution of ERIs had no differences; however ERIs were more common in younger patients (47.6 vs. 33.4, p<.001). ERIs rates were significantly higher in an ER compared to ICU/OR (13% vs. 7.7%, p=.03). Non-stroke patients had higher rates of ERIs compared to stroke related patients (17% vs. 6.5%, p<.001). Moreover, 153 out of the overall 704 patients required a conversion to VP shunt (21.7%) from which 26.8% (n=41) patients had a diagnosed ERI, while only 6.7% (n=37) of the 551 patients that did not require a conversion to VP shunt had reported a ERI (Table 3). The goal of this study was to focus on the environment where EVDs are placed. The fact that we found several variables associated to ERIs, we assessed if any of these variables were over-represented in a particular environment (ER or ICU/OR). Neither females, younger patients nor non-stroke cases that had higher rates of ERIs were over-represented in any setting (Table 4). 

**Table 3:** Rates of EVD infections following placement in the ER and ICU External ventricular drainage (EVD), emergency room (ER), intensive care unit/operating room (ICU/OR), ventriculo-peritoneal (VP)

Parameters		EVD related infection				
		No		Yes		P Value
		Count (n=626)	N %	Count (n=78)	N %	
Gender	M	341	89.30%	41	10.70%	0.75
	F	285	88.50%	37	11.50%	
Age (mean and SD)		47.65	23.62	33.49	25.82	<0.001
Place	ER	387	87%	58	13%	0.03
	ICU	239	92.30%	20	7.70%	
Stroke related	No	254	83%	52	17%	<0.01
	Yes	372	93.50%	26	6.50%	
Conversion to VP Shunt	No	514	93.30%	37	6.70%	<0.01
	Yes	112	73.20%	41	26.80%	

**Table 4:** Distribution of variables between ER and ICU setting External ventricular drainage (EVD), emergency room (ER)), intensive care unit/operating room (ICU/OR), ventriculo-peritoneal (VP)

Parameters		Place				
		ER		ICU/OR		P Value
		Count (n=451)	N %	Count (n=259)	N %	
Sex	F	202	62.0%	124	38.0%	0.427
	M	249	64.8%	135	35.2%	
Age (mean and SD)		44.14	24.93	49.16	22.91	0.078
Stroke	No	206	66.9%	102	33.1%	0.088
	Yes	242	60.7%	157	39.3%	
Conversion to VP Shunt	No	344	62.1%	210	37.9%	0.181
	Yes	104	68.0%	49	32.0%	

Finally, from the 26 stroke-diagnosis EVDs that developed ERIs, 6 (23.1%) required conversion to VP shunt. In the non-stroke group, 35 out of the 52 patients that developed an ERI (67.3%) required conversion to VP shunt which is statistically significant compared to the 72 patients (28.3%) out of 254 non-stroke patients without ERI that required VP shunt (p<.001) (Table 5).

**Table 5:** Conversion of EVD to VP shunt after ERI n stroke and non-stroke related patients External ventricular drainage (EVD), emergency room (ER), intensive care unit/operating room (ICU/OR), ventriculo-peritoneal (VP)

Parameter		Stroke									
		No					Yes				
		EVD related infection									
		No		Yes		P Value	No		Yes		P value
		Count (n=254)	N %	Count (n=52)	N %		Count (n=372)	N %	Count (n=26)	N %	
Conversion to VP Shunt	No	182	71.70%	17	32.70%	<0.001	332	89.20%	20	76.90%	0.057
	Yes	72	28.30%	35	67.30%		40	10.80%	6	23.10%	

## Discussion

Our study focused on the comparison of EVD related infections (ERIs) rates between Emergency Room (ER) and Intensive Care Unit (ICU)/ Operating Room (OR) setting among stroke and non-stroke related patients from all ages that were treated from January 2010 until June 2018 in our single academic center that includes the main adult hospital and the children division. We based this study on the introduction of a new protocol from July 1st 2015, which required to change the location of EVD placement from ER to ICU/OR. Although iatrogenic factors contributing to EVD infections are not well characterized yet, we aimed to study how the environment where EVDs are placed can be associated with ERIs in a large sample [1]. There was no difference over these years in the equipment used, protocol of steps educated, nor bundling of equipment during this time.

Our rates of ERIs are within the previous rates that have been reported [12]. However, we had a significant drop of ERIs after the new protocol was applied. ER rates of infections went from 13% to 7.7% in the ICU/OR. Although, the literature of safest environment to place an EVD is still inconsistent, some studies have concluded that the Operating Room (OR) is the optimal setting for EVD placement but nevertheless, other authors have suggested lower risk of ERIs in ICU setting [3-15,16]. Moreover, outcomes and complications between OR, ICU and ER have several confounders as the decision of a particular setting to place an EVD can be related to the status of the patient at initial diagnosis and requirement of an emergent procedure [1]. To our knowledge, this is the first large-sample single center study that made the decision of setting based on a protocol and not on other factors like the status of the patient at admission. Therefore, our cohorts of ERIs in ER and ICU/OR setting comprise a more heterogeneous sample. Moreover, we identified higher rates of ERIs in younger patients, non-stroke cases and in patients that required conversion to VP shunt. However, the majority of the current evidence indicates that age is not an independent predictor factor for ERI. Regarding the diagnosis or indication for EVD placement related to ERIs, there’s still inconsistent evidence about how much different types of brain injuries contribute to susceptibility to infections (by inducing immune modulation or due to the different management, protocols, severity and complications) between stroke and non-stroke cases and also, within each category [6,17-19].

For the purpose of this study, we identified whether there was any difference in the distribution of variables between ER and ICU/OR setting. We had similar distribution of sex, age, diagnosis and required conversion of EVD to VP shunt in both settings. We found younger patients and non-stroke cases are more likely to have ERIs, neither of these populations were overrepresented in the ER or ICU/OR sample. Finally, our study highlights the relation between ERIs and further conversion of EVD to VP shunt, unlike other studies that haven’t found different rates of conversion to VP shunt in patients with CSF infection [1,18]. However, this relation between ERIs and further conversion to VP shunt seems to be limited to non-stroke cases according to our results. Similar previous studies had pointed non-stroke causes such as tumors and obstructive hydrocephalus are more likely to require a VP shunt, and within this population, CSF infection may play an important role as predictor on the long-term care of patients [19]. But nevertheless, most of these studies have been done in pediatric populations or in small adult patient’s samples, making comparison difficult to address properly.

The findings of this study have to be seen in light of some limitations inherent in a single-center retrospective design. Therefore, our study is subject to biases and confounding factors that may have influenced our study estimates. First of all, comparisons with previous studies is limited due to different inclusion criteria, methods, judgement and definitions of EVD related infections in different reports. Moreover, most of the studies have split the place of EVD placement between ER, ICU and OR [12,20]. Our study is limited to differences between ER and ICU/OR setting as we aimed to demonstrate if ER environment can be considered a iatrogenic factor for ERIs. Furthermore, all our patients underwent EVD placement with non-antibiotic impregnated EVD catheters making comparison with other centers even more difficult and finally, our different rates of ERIs between environments are not supported by different types of microorganisms in CSF cultures. As the experience or training of the resident in charge of the EVD placement was not registered in our data, the contribution of the procedure experience was not assessed; however, the large sample considered in this study might have reduced the variability of this factor. Nevertheless, we consider these findings important to be communicated with the neurosurgical community in order to improve outcomes, prevent complications and contribute to the literature of one of the most common procedures in the neurosurgical field.

## Conclusions

Our study evidenced a relation between EVD related infections and the location where these procedures are performed. After the application of a new protocol in our single center institution that changed EVD placement from the Emergency Room to the Intensive Care Unit or the Operating Room, the ERIs rates significantly reduced among patients from all ages and independent of the type of brain injury. Moreover, our results highlight the relation between ERIs and further requirement of conversion of an EVD to a VP shunt.
